# Death of Mr. Liston

**Published:** 1848-02

**Authors:** 


### DEATH OF MR. LISTON.

Our correspondent at Paris writes us, December 14th: "The news of Mr. Liston's death has just reached us, and, as you may imagine, has saddened the faces of us all. No particulars have been received, except that he died after an illness of three weeks, of hemorrhage of the lungs, and that science is in mourning wherever the sad intelligence has spread. It is reported that the hemorrhage was connected with aneurism of the aorta." Our correspondent promises us in his next letter a fuller account of his case. Mr. Liston was as favorably known as any European surgeon to the profession of our country, and the cordiality of his manners endeared him to the American student in London. Referring to this quality our correspondent remarks: "No stranger ever treated me with the cordiality, and, I may say, the affection, that Mr. Liston did. I looked forward to the coming spring with double pleasure, since I hoped to spend a portion of it in his society."

